# Immunological risk stratification and tailored minimisation of immunosuppression in renal transplant recipients

**DOI:** 10.1186/s12882-020-01739-3

**Published:** 2020-03-11

**Authors:** Mysore K. Phanish, Richard P. Hull, Peter A. Andrews, Joyce Popoola, Edward J. Kingdon, Iain A. M. MacPhee, for the South West Thames Renal Transplantation Network

**Affiliations:** 1 South West Thames Renal and Transplantation Unit, St Helier Hospital, Epsom and St Helier University Hospitals NHS trust, Carshalton, UK; 2 SW Thames Institute for Renal Research, St Helier Hospital, Carshalton, Surrey SM5 1AA UK; 3 Renal Unit, King’s College Hospitals NHS Foundation Trust, London, UK; 4 Renal Medicine and Transplantation, St George’s University Hospitals NHS Foundation Trust, London, UK; 5 Renal Unit, Epsom and St Helier University Hospitals NHS Trust, Carshalton, UK; 6 Sussex Kidney Unit, Brighton and Sussex University Hospitals NHS Trust, Brighton, UK

**Keywords:** Corticosteroid-withdrawal, Basiliximab, Mycophenolate mofetil, Renal transplantation, Tacrolimus

## Abstract

**Background:**

The efficacy and safety of minimisation of immunosuppression including early steroid withdrawal in kidney transplant recipients treated with Basiliximab induction remains unclear.

**Methods:**

This retrospective cohort study reports the outcomes from 298 consecutive renal transplants performed since 1st July 2010–June 2013 treated with Basiliximab induction and early steroid withdrawal in low immunological risk patients using a simple immunological risk stratification and 3-month protocol biopsy to optimise therapy. The cohort comprised 225 low-risk patients (first transplant or HLA antibody calculated reaction frequency (CRF ≤50% with no donor specific HLA antibodies) who underwent basiliximab induction, steroid withdrawal on day 7 and maintenance with tacrolimus and mycophenolate mofetil (MMF), and 73 high-risk patients who received tacrolimus, MMF and prednisolone for the first 3 months followed by long term maintenance immunosuppression with tacrolimus and prednisolone. High-risk patients not undergoing 3-month protocol biopsy were continued on triple immunosuppression.

**Results:**

Steroid withdrawal could be safely achieved in low immunological risk recipients with IL2 receptor antibody induction. The incidence of biopsy-proven acute rejection was 15.1% in the low-risk and 13.9% in the high-risk group (including sub-clinical rejection detected at protocol biopsy). One- year graft survival was 93.3% and patient survival 98.5% in the low-risk group, and 97.3 and 100% respectively in the high-risk group. Graft function was similar in each group at 1 year (mean eGFR 61.2 ± 23.4 mL/min low-risk and 64.6 ± 19.2 mL/min high-risk).

**Conclusions:**

Immunosuppression regimen comprising basiliximab induction, tacrolimus, MMF and prednisolone with early steroid withdrawal in low risk patients and MMF withdrawal in high risk patients following a normal 3-month protocol biopsy is effective in limiting acute rejection episodes and produces excellent rates of patient survival, graft function and complications.

## Background

Current immunosuppression regimens now produce low rates of early rejection and high rates of graft survival in the early years following renal transplantation. Most of the immunosuppressive regimes used worldwide utilise long-term triple immunosuppression with Tacrolimus, Prednisolone and Mycophenolate mofetil. Maintenance immunosuppression without corticosteroids is usually only considered if the induction agent is a lymphocyte depleting antibody such as anti-thymocyte globulin (ATG) or Alemtuzumab (Campath). Minimising corticosteroid exposure has well-known clinical benefits, but the clinicians are often reluctant to withdraw corticosteroids in early post-transplant period in the absence of lymphocyte depleting antibody induction.

The use of induction antibodies has reduced the rates of early acute rejection and graft loss in the first year after transplantation [1–6]. Tacrolimus is now established as the primary agent for maintenance immunosuppression [7–9]. The ELITE-Symphony trial, which combined low dose tacrolimus with anti-IL-2 receptor antibody induction and mycophenolate mofetil (MMF) maintenance, defined the current standard of treatment. This combination achieved superior outcomes in terms of graft function, survival and biopsy-proven acute rejection (BPAR) at 1 year which was maintained at 3 years compared to ciclosporin or sirolimus based regimens [10, 11]. What remains uncertain is whether the relatively high doses of MMF used in this study are necessary to maintain long-term graft function, and whether long-term steroid use is necessary to achieve these good outcomes in lower immunological risk patients. The ATLAS studies have indicated that steroid-free tacrolimus based regimens can achieve good long term outcomes [12–14]. Lymphocyte depleting antibodies such as anti-thymocyte globulin (ATG) and Alemtuzumab (Campath) have been used to achieve steroid avoidance/early withdrawal but use of these drugs carry a risk of over-immunosuppression [6, 15–17]. For recipients at higher immunological risk, there are still major challenges to overcome such as higher rates of acute rejection and poor graft survival [18–20].

With these factors, challenges and available evidence at the time in mind, our transplant network instituted a new immunosuppression regimen in July 2010 that stratified patients according to immunological risk, as defined below. All patients received induction with basiliximab, low-risk patients underwent steroid withdrawal at day 7, high-risk patients continued tacrolimus based triple regimen and both risk groups underwent dose reduction of MMF at day 30. Protocol biopsy was performed at day 90 to allow for specific tailoring of immunosuppression. The primary objectives of this protocol were minimisation of immunosuppressive burden while achieving acceptable rates of rejection, steroid withdrawal in low immunological risk patients and the avoidance of lymphocyte depleting antibodies. This manuscript describes the excellent short-term outcomes of this risk stratification regimen.

## Methods

### Patients

This was a retrospective cohort analysis. All adult recipients who received a renal transplant at our surgical centre between 1st July 2010 and 31st June 2013 and who had received a minimum of 3 months follow-up were included (225 low-risk and 73 high-risk patients). The donor pool comprised donation after brainstem death (DBD), donation after circulatory death (DCD) and living donors including altruistic and paired exchange scheme kidneys. Recipients from ABO-incompatible, HLA-antibody incompatible (Flow cytometry crossmatch positive) living donations were excluded. Recipients positive for donor-specific HLA antibodies (DSA) with or without crossmatch positivity were also excluded. No other exclusion criteria were applied. All waitlisted patients had their HLA antibody status assessed regularly by Luminex assays (Life Technologies Ltd., Paisley, UK) and after any sensitising event such as blood transfusion. HLA antibody detection and cross match at the time of transplantation were done using a combination of complement-dependent cytotoxicity (CDC) crossmatch, flow cytometry and Luminex assays. If HLA antibodies were detected by screening Luminex screening, antigen specificities were further assessed by single antigen beads.

All patients underwent transplantation at St George’s Hospital followed by early repatriation to their local renal centre. The transplant network comprises St George’s University Hospitals NHS Foundation Trust Renal Unit; the South West Thames Renal and Transplantation Unit based at Epsom and St Helier University Hospitals NHS Trust, Surrey, UK; and the Sussex Kidney Unit based at Brighton and Sussex University Hospitals NHS Trust, Brighton, UK.

### Immunosuppression regimen

Patients were stratified prior to transplantation according to immunological risk. Low-risk patients were first transplant recipients with a CRF < 50%. High-risk patients were those receiving their second or subsequent transplants or with CRF ≥50%. There was no stratification based on organ donor type, degree of HLA mismatch and recipient ethnicity. All patients received induction therapy with basiliximab (Simulect) 20 mg given intravenously perioperatively and on day 4, and peri-operative methylprednisolone 500 mg intravenously and were maintained on tacrolimus, administered pre-operatively at a dose of 0.075 mg/kg followed by 0.075 mg/kg twice daily (adjusted to 0.15 mg/kg for those of sub-Saharan African or African-Caribbean origin). Target tacrolimus whole blood trough concentrations measured by immunoassay were 10–15 ng/mL day 0–30, 8–12 ng/mL from day 31–90 and 5–8 ng/mL from day 91. MMF was dosed at 1000 mg twice daily for days 0–30 with reduction to 500 mg twice daily from days 31–90. In the low-risk group, oral prednisolone 20 mg once daily was administered from day 1–7 and stopped on day 8. In the high-risk group, prednisolone was continued with a reducing regimen to 5 mg once daily by day 43.

Acute rejection (at least Banff grade I) was treated with intravenous methylprednisolone 500 mg daily for 3 days. Following an episode of rejection, the tacrolimus concentration was increased to the upper end of the therapeutic range, MMF was added if not previously prescribed at a dose of 1 g twice daily and MPA exposure was optimised (using estimation of AUC as employed in the FDCC study [21]) in some cases. Prednisolone was started or increased to 20 mg once daily for 1 week with a reduction to 5 mg daily or the pre-rejection steroid dose (whichever was higher) through a 4-week taper. Patients with steroid-resistant/vascular rejection underwent repeat biopsy and received treatment with antithymocyte globulin (ATG/Thymoglobulin). There was no patient with an acute antibody mediated rejection (Acute AMR) in this cohort. All patients treated for rejection underwent a further biopsy 3 month after rejection to guide long-term immunosuppression. High-risk patients for CMV (D + R-) were given valganciclovir prophylaxis for 100–200 days. Routine CMV and BKV viral load-based surveillance was not performed. In addition to routine histological analysis, all renal biopsies were stained for SV40 large T-antigen (for BKV) and C4d by immunohistochemistry. Rejection was classified according to the Banff 1997 grading system.

Protocol biopsies were performed at 3 months after transplantation to exclude subclinical rejection prior to any modifications of immunosuppression and to screen for other pathologies such as BK virus nephropathy, IFTA (Interstitial fibrosis and tubular atrophy) and evidence of calcineurin inhibitor toxicity. Sub-clinical rejection detected on protocol biopsy (Banff 1A or above) was treated as above with a further biopsy 3 months after rejection. In the low-risk group, a normal biopsy resulted in a switch from MMF to azathioprine 1.5 mg/kg once daily. In the high-risk group, patients with a normal biopsy had a reduction then cessation of MMF after 7 days. Patients unable to tolerate steroid therapy or with steroid-related complications stopped prednisolone and continued tacrolimus and MMF.

The data presented in this study was routinely collected as part of our annual transplant audit hence the need for ethics approval was waived by the national health research authority (HRA, UK). The study was registered with trust (Epsom and St Helier University Hospitals NHS Trust) research and development board and approved by HRA, England (REC ref.: 17/LO/1352, IRAS Id-270,665).

### Statistical analysis

Statistical analysis was performed in GraphPad Prism version 8 and Microsoft Excel 2007. Patient demographics were compared using the Chi-square statistic and unpaired t test (to compare means and proportions) for categorical and non-categorical variables respectively. Patient and graft survival and freedom from BPAR were analysed using Kaplan-Meier survival with the Log-rank (Mantel-Cox) test used to compare the groups. Graft function was estimated using the Chronic Kidney Disease Epidemiology Collaboration (CKD-EPI) formula. Comparison of CKD-EPI eGFR at each timepoint was done by 1-way ANOVA with multiple comparison tests to assess pairwise comparisons. To handle missing data, we also analysed eGFR at each time point using a repeated measures ANOVA with mixed-effects model (REML). Analysis of protocol biopsy risk factors for rejection and NODAT rates were analysed using a two-sided Fisher’s exact test as part of contingency table analysis.

## Results

### Patient demographics and tacrolimus blood concentrations

**Fig. 1:**
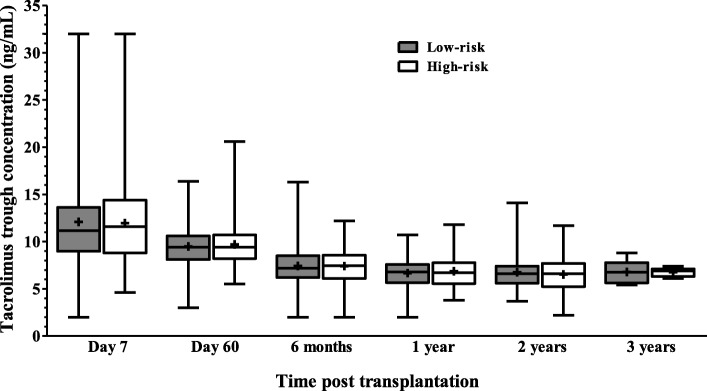
Tacrolimus trough levels (ng/mL) obtained over study period. Levels measured by liquid chromatography-tandem mass spectrometry for each immunological risk group are given (line at median, upper and lower quartiles, range, cross indicates mean)

Demographics of the two groups of patients were similar (except for higher proportion of LD and low proportion of DCD donors in high risk group) with most patients being male and of white ethnicity with comparable HLA MM (Table 1) with a median FU of 610 days. Tacrolimus blood concentrations achieved is shown in Fig. 1.

**Table 1:** Baseline characteristics of transplant recipients

	Low-risk group (***n*** = 225)	High-risk group (***n*** = 73)	***p***-value
Sex			0.57
Male	144 (64)	44 (60.3)	
Female	81 (36)	29 (39.7)	
Ethnicity			0.32
White	162 (72.0)	60 (82.2)	
S. Asian	38 (16.9)	8 (11.0)	
Black	23 (10.2)	4 (5.5)	
Others	2 (0.9)	1 (1.4)	
Age			0.24
Mean ± SD	50.2 ± 13.6	51.6 ± 14.1	
Range	19.4–74.2	20.0–73.1	
Donor type			
Living	89 (39.6)	38 (52.1)	0.06
DBD	95 (42.2)	31 (42.5)	0.96
DCD	41 (18.2)	4 (5.5)	0.008
Length of follow up (days)			
Mean ± SD	539.7 ± 20.4	691.5 ± 38.5	< 0.01 (95%CI
Range	51–1139	139–1181	144–158)
Primary renal diagnosis			
Diabetes mellitus	16 (7.1)	1 (1.4)	0.07
Glomerulonephritis	76 (33.8)	24 (32.9)	0.88
Pyelonephritis	21 (9.3)	8 (11.0)	0.67
Hypertension	13 (5.8)	3 (4.1)	0.57
Autosomal dominant polycystic kidney	37 (16.4)	11 (15.1)	0.79
Renal vascular disease	12 (5.3)	2 (2.7)	0.36
Other	21 (9.3)	12 (16.4)	0.09
Uncertain aetiology	29 (12.9)	12 (16.4)	0.45
HLA			
MM	3.17 (1.55)	3.12 (1.24)	0.22

Values are expressed as mean ± SD or n (%)

### Patient and graft survival

**Fig. 2:**
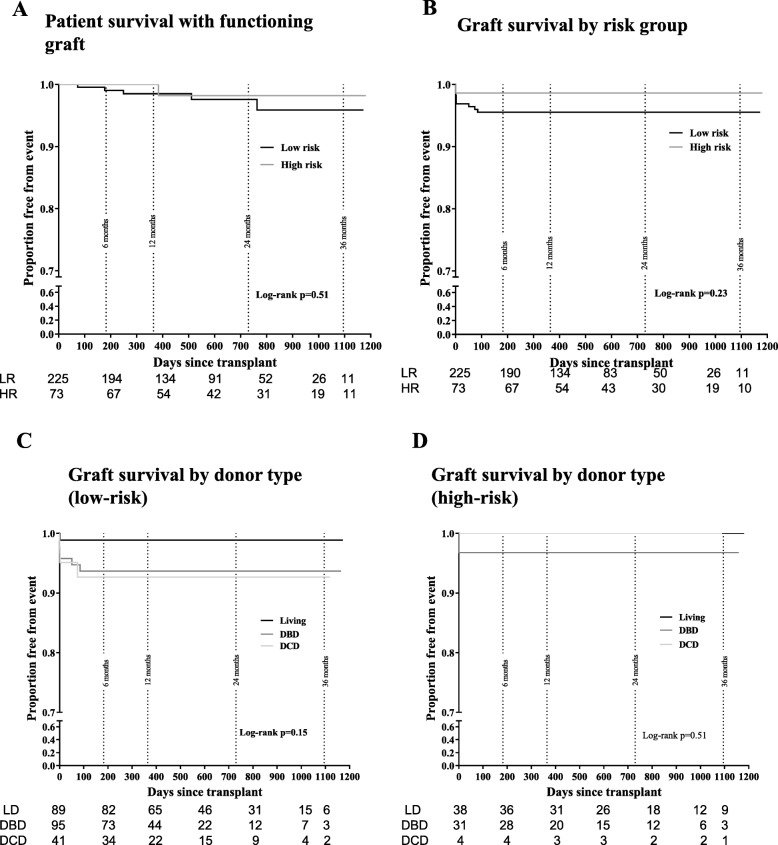
Patient and graft survival (Kaplan-Meier estimates) and function. **a** Patient survival with a functioning graft. **b** Graft survival by immune risk group. **c** Graft survival by donor type in low-risk and **d** high-risk patients. Numbers at risk at various time points is given below each graph. Graph beyond 400 days should be interpreted with caution due to low ‘numbers at risk’

One-year overall survival rates for patients were 98.5% in the low-risk and 100% in the high-risk group. These rates were maintained at 2 years (97.6% low-risk, 98.2% high-risk). No difference was seen in patient survival between the two groups (*p* = 0.51 by log-rank test) (Fig. 2a). There were five deaths in patients from the low-risk cohort and one death in the high-risk group (Table 2). Overall graft survival for the follow-up period was 93.3% in the low-risk and 97.3% in the high-risk group. There was no significant difference between the two risk groups (*p* = 0.23 by log-rank test) (Fig. 2b). One-year overall graft survival was 93.8% (95.1% censored for death) in the low-risk group and 98.6% (98.6% censored for death) in the high-risk group. There was no difference in graft survival by donor type in each of the groups (*p* = 0.15 by log-rank test in low-risk, *p* = 0.51 by log-rank test in high-risk cohort) (Figs. 2c & d). In these Kaplan-Meier estimates, graph beyond 400 days should be interpreted with caution due to low ‘numbers at risk’.

**Table 2:** Causes of graft loss

	Low-risk group (*n* = 225)		High-risk group (*n* = 73)	
	n	%	n	%
Primary non-function	3	1.3	0	0
Early vascular complications including thrombosis	7	3.1	1	1.4
Death with functioning graft	5	2.2	1	1.4
Sepsis	1	0.4	0	0

### Graft function

**Fig. 3:**
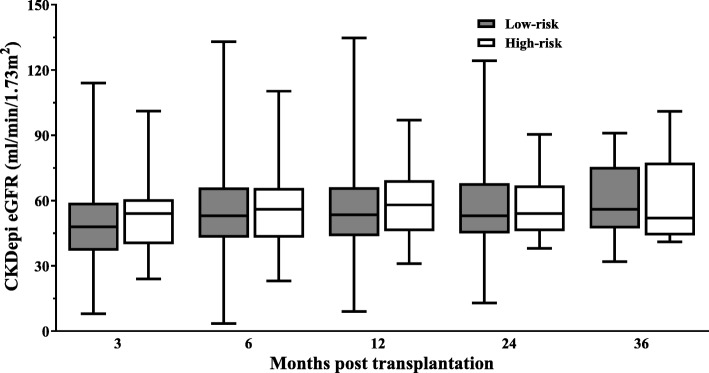
Graft function between low and high risk groups as assessed by calculated CKD-Epi eGFR at 3, 6, 12, 24 and 36 month time points. Comparisons at each time point non-significant by one-way ANOVA with Bonferroni multiple comparisons test (P 0.92, 3 m; P 0.99, 6 m; P 0.99, 12 m; P 0.99, 24 m; P 0.99, 36 m) and repeated measures ANOVA with mixed effects model analysis

Graft function was assessed at 3, 6, 12, 24 and 36 months after transplantation. There was no statistical difference (analysed by one-way ANOVA with multiple comparison test and repeated measures ANOVA with mixed effects model) between the mean eGFR in the low and high-risk groups at any of the time points (Fig. 3). The mean eGFR at 6 months was 59.4 ± 22.7 mL/min in the low-risk group (*n* = 203) and 61.8 ± 19.0 mL/min in the high-risk group (*n* = 72). Graft function was preserved at 1 year (mean eGFR 61.2 ± 23.4 mL/min low-risk and 64.6 ± 19.2 mL/min high-risk and at 2 years (mean eGFR 61.2 ± 22.9 mL/min low-risk and 61.6 ± 16.1 mL/min high-risk.

### Rejection episodes

**Fig. 4:**
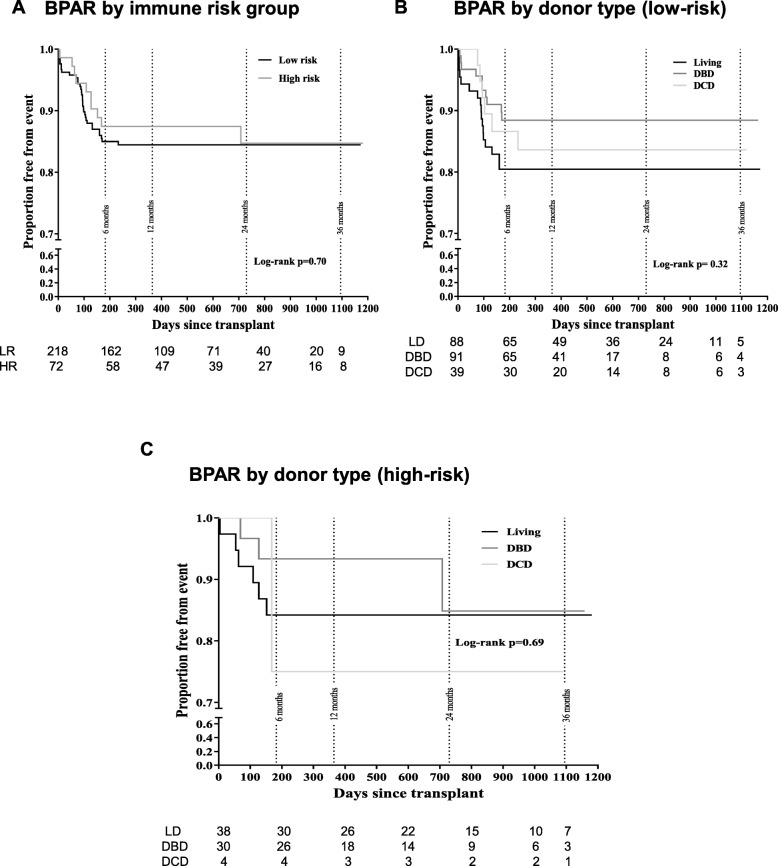
Biopsy-proven acute rejection (BPAR) rates. Kaplan-Meier estimates for BPAR by immune risk group (**a**), donor type in low-risk (**b**), and in high-risk (**c**) recipients. Numbers at risk at various time points is given below each graph. Graph beyond 400 days should be interpreted with caution due to low ‘numbers at risk’

There was no significant difference between the rate of BPAR in the two treatment groups (*p* = 0.70 by log-rank test) (Fig. 4a). There was no difference in BPAR (expressed as survival free from rejection) when analysed by donor type in either group; *p* = 0.32 by log-rank test in low-risk (Fig. 4b), *p* = 0.69 by log-rank test in high-risk group (Fig. 4c). Due to very small number of DCD donors in high risk group, we analysed DBD and LD separately in this group (no significant difference between the groups, Log rank *P* = 0.49).

Within the low-risk group, there were 33 BPAR episodes of Banff 1A or above (15.1%), of which only one occurred after 6 months (Table 3). Of these 33 episodes, 21 (9.6% of the low-risk group) were identified at biopsies performed for clinical indications and 12 (5.5% of the low risk group) were identified in protocol biopsies (Table 3). There were 10 BPAR episodes in the high-risk group, a rate of 13.9% overall for the study period (Table 3). Of these 10 episodes, 8 (11.1% of the high-risk group) were identified at clinically indicated biopsies and 2 (2.8% of the high-risk group) in protocol biopsies.

**Table 3:** Biopsy-proven acute rejection rate (BPAR)

Group	n in group	Total BPAR		CI-BPAR		PB-BPAR	
		n	%	n	%	n	%
Low-risk	218	33	15.1	21	9.6	12	5.5
High-risk	72	10	13.9	8	11.1	2	2.8
	Low-risk			High-risk			
Donor type	n in group	BPAR		n in group	BPAR		
		n	%		n	%	
Living	88	17	19.3	38	6	15.8	
DBD	91	10	11	30	3	10	
DCD	39	6	15.4	4	1	25	

*Abbreviations*: *BPAR* Biopsy-proven acute rejection, *CI-BPAR* Clinically-indicated BPAR, Protocol biopsy identified BPAR, *DBD* Donation after brain death, *DCD* Donation after Circulatory death

### Protocol biopsy, azathioprine switch and infectious complications post transplantation

In the low-risk group, 171 patients underwent protocol biopsy (77.5% of group). This identified 12 cases (7%) of mild to moderate rejection, graded Banff IA–IIA. In the high-risk group, 43 (59.7%) patients underwent protocol biopsy and 2 cases (4.6%) of mild BPAR (1A) were identified. Patients from high-risk group not undergoing protocol biopsy were continued on triple immunosuppression. Although there was a trend towards higher protocol biopsy detected rejection rates in low risk (Early steroid withdrawal) patients, there was no statistically significant difference between the two groups (p 0.6, Chi square with Fisher’s exact test). At the time of protocol biopsy, 10 of the 12 low-risk patients with rejection had a eGFR < 60 mL/min/1.73m^2^, but this did not reach significance compared to the overall cohort who underwent protocol biopsy (Fisher’s exact test *p*-value = 0.06, RR 3.99 95% CI 0.93–17.11). In the high-risk group, out of 2 patients who had rejection detected on protocol biopsy, one had an eGFR of < 60 and one > 60. In addition to sub-clinical rejection, protocol biopsy identified two cases of BKV nephropathy. Eighty low risk patients underwent azathioprine switch which was safe with no BPAR episodes identified following the change. Graft function was maintained at with a mean eGFR of 58.9 ± 20.1 mL/min at 3 months pre-conversion and a mean eGFR of 66.7 ± 22.6 mL/min at 6 months (*n* = 79) and 68.0 ± 24.3 mL/min at 1-year post conversion (*n* = 61). There were low rates of clinically significant viral infections. Eight patients (2.8% of total number of patients) developed CMV disease. Three patients were positive for CMV IgG pre-transplantation and five were CMV-negative recipients who received kidneys from CMV-positive donors. Six cases of BK polyoma virus infection were identified (2.1%). All were recipients of deceased donor kidneys, three were low-risk, and three were high-risk patients. Two of the cases in high-risk patients were diagnosed by protocol biopsy. No grafts were lost because of viral infection.

### New-onset diabetes after transplantation (NODAT, post -transplant diabetes mellitus)

Seventeen cases of NODAT were identified, all occurring in the first three months after transplantation. The overall rate of NODAT was 5.9% with nine cases occurring in the low-risk group (4.12% of the low-risk group) and eight cases in the high-risk group (11.1% of the high-risk group). The incidence of NODAT was lower in the steroid withdrawal (low immunological risk) group (Fisher’s exact test *p*-value = 0.041, RR 2.69 95% CI 1.08–6.72).

## Discussion

In this report, we present short-term outcomes of our risk-stratified transplant immunosuppression regimen demonstrating acceptable rates of BPAR and high rates of patient and graft survival. Our data suggest that early steroid withdrawal can be undertaken successfully in appropriately selected recipients in real-world clinical practice outside of a randomised controlled trial without requiring lymphocyte depleting antibody induction. The protocol used is easy to implement, practical and clinically driven.

Corticosteroid induced complications such as NODAT, weight gain and osteoporosis have driven attempts to minimise steroid-exposure [22, 23]. While there are concerns that early steroid withdrawal may increase the incidence of acute rejection [24], a number of studies report good long term transplant outcomes alongside major benefits in reducing cardiovascular risk factors such as hyperlipidaemia [25–27]. IL-2 receptor antagonist induction has allowed earlier steroid withdrawal to a point where corticosteroids have been confined to use at induction [28–31]. ATLAS examined the feasibility of a maintenance steroid free regimen in comparison with a tacrolimus-based triple regimen in low-risk patients. Though there were relatively high rates of biopsy-proven acute rejection in the first 6 months, 80% of patients were still steroid free at 6 months [14]. Critically, there were no differences in patient and graft survival or graft function between the steroid free and standard triple regimen groups up to 3 years after transplantation [12–14].

Our risk stratification driven immunosuppression with predominantly two drug long-term immunosuppression was designed to balance the risk of over-immunosuppression against the risk of rejection and long-term graft damage. The majority of cases of rejection were mild to moderate and confined to the first 6 months after transplantation. The 1 year BPAR rate was very similar to those outcomes achieved by randomised control trials that included basiliximab-based regimens [15, 17, 28, 31] and better than outcomes from some low-risk steroid minimisation studies where rates have been as high as 31.8% [6, 13, 16]. A sizeable proportion of kidneys came from DCD donors in the low-risk group suggesting that such kidneys can have excellent outcomes approaching those of living and DBD grafts with standard immunosuppression used in our protocol. Patient and graft survival were high in both cohorts and there was no influence from donor type although we acknowledge very small number of patients with DCD donors in high immunological risk group.

Graft function is a critical outcome, particularly as lower GFR at 1 year after transplantation is associated with graft loss [10, 32, 33]. Using our protocols, graft function in both the low-risk (early steroid withdrawal) group and high-risk group was well preserved between 6 months and 2 years, in line with other studies, [12, 16, 31]. Three-month protocol biopsies added value to the management of our patients by detecting subclinical rejections (as reported before in published literature) [34] and BK virus nephropathy. Although there was a tendency towards higher rates of rejections detected on protocol biopsy in steroid withdrawal (low risk) group, there was no statistically significant difference in the rate of rejections detected on protocol biopsy between the low immunological risk (steroid withdrawal) group and high immunological risk group therefore, based on our results, protocol biopsies can be justified in both cohorts. However, we do acknowledge that with a larger cohort and appropriate statistical power to detect the difference, this difference in incidence of rejections detected on protocol biopsy could reach statistical significance with higher rates in steroid withdrawal cohort. In this study, we could not identify any statistically significant factors that predicted whether rejection might be identified at 3 months to individualise our biopsy strategy. However, out of 14 rejections detected on protocol biopsies (12/171 in low risk group and 2/43 in high risk group, majority (11 out of 14, 78%) had an eGFR of < 60 ml/min at the time of 3-month protocol biopsy (10 in low risk group and 1 in high risk group). Although this did not achieve statistical significance, an eGFR of < 60 at 3 months could be used as a guide to tailor protocol biopsies taking in to account invasive nature of the procedure and potential risks involved. Absence of rejection on protocol biopsies allowed us withdrawal of mycophenolate mofetil in high immunological risk patients resulting in overall reduction in long term immunosuppression, low CMV rates without increase in rejection rates. Our 12 month rate of steroid-free therapy (73%) was comparable to majority of the published literature where 60–70% compliance has usually been achieved [12, 15, 16, 28, 31]. Initiation of steroid therapy was at the discretion of the treating physicians at each follow up centre and usually followed an episode of BPAR or withdrawal of MMF/Azathioprine due to adverse effects such as leukopenia and gastrointestinal side effects (in case of MMF).

Our dosing regimen for MMF was based on studies indicating that plasma concentrations of mycophenolate increase over time in patients with renal impairment after transplantation [35]. Rather than adopting therapeutic drug monitoring, we based our dosing regimen on doses predicted to achieve optimal exposure in the FDCC study [21]. It has been argued that reduction of MMF doses below 2 g daily leads to under-exposure with increased rates of acute rejection [36]. This view is pharmacokinetically illogical in tacrolimus-treated patients and a systematic reduction in MMF dose to 500 mg twice daily in our cohort delivered a very acceptable rate of acute rejection. Conversion from MMF to azathioprine at 3 months was found to be safe in the low-risk group, with no associated rejection episodes, better tolerated and resulted in maintained graft function. This is of particular relevance in parts of the world where cessation of medication due to cost is a major reason for graft failure [37].

Our strategy aimed to minimise complications associated with immunosuppression. The incidence of NODAT was lower in the low-risk group, confirming the beneficial effect of steroid withdrawal on the incidence of NODAT. Studies using steroid-free protocols, where reported, mostly demonstrate rates of CMV disease of between 5 and 10.2% [6, 14–16]. The low levels of CMV disease and BK nephropathy in our cohorts reflect our strategy of avoiding over-immunosuppression.

Hanaway et al. [6] compared different antibody induction regimes with early corticosteroid withdrawal. The rejection rates were lower in patients receiving alemtuzumab (3%) compared to conventional induction (15%), and rejection rates were 10% in low risk patients in the alemtuzumab group and 22% in the basiliximab group. We used basiliximab for both our low and high-risk groups, achieved a successful steroid withdrawal in the low risk group (which included patients with a PRA of up to 50%) and have avoided the potential long-term risks associated with the use of lymphocyte depleting antibodies for induction. National statistics published by ODT, UK show that we have excellent 5y patient and graft survival rates (available in public domain, https://www.odt.nhs.uk/, organ specific reports). We believe that our rejection rate of 13–15% provides a good balance between over and under-immunosuppression with excellent short and medium-term graft outcomes. However, it must be noted that our high immunological risk patients did not have donor specific HLA antibodies (DSA) at the time of transplantation and therefore, one could argue that this group of patients should be classed as intermediate risk rather than high risk. Our approach may or may not be applicable to recipients with DSAs. Whilst MMF withdrawal following normal protocol biopsy in high risk patients and early steroid withdrawal in low risk patients did not lead to adverse outcomes over short term follow up period, these patients may develop DSAs over a period and therefore, long term (> 10 years) patient and graft outcomes in this cohort needs to be analysed before making recommendation on appropriateness of this protocol for long term management. A limitation of the study is that mean follow-up was 2 years and therefore, long term data needs to be analysed to confirm if favourable outcomes observed in this short and intermediate term analysis persist over a longer period of time post-transplant. Other limitation of the study is small sample size in the high immunological risk group and that a proportion of high-risk patients (43/73, 40%) did not undergo protocol biopsy and continued on triple immunosuppression, which could have a bearing on the results shown. Main objective of this work is to report outcomes of our transplant immunosuppression strategies in low and high immunological risk groups and we acknowledge that the groups are immunologically dissimilar and as such not directly comparable. A randomised prospective trial with analysis of short- and long-term outcomes will be needed to make firm recommendations.

## Conclusions

IL-2 receptor antagonist induction, tacrolimus and MMF based immunosuppressive regimen with early steroid withdrawal in low-risk patients is feasible in clinical practice and results in acceptable rates of acute rejection with maintenance of renal function and low levels of graft loss without requiring induction with a T cell depleting antibody. In our high-risk patients, a triple therapy-based regimen with IL-2 receptor antagonist induction was safe and produced good outcomes and excellent graft function when compared to our low-risk cohort and other high-risk patient cohorts reported in literature. Maintenance immunosuppression following a 3-month protocol biopsy of either Tacrolimus/Azathioprine in low-risk group and Tacrolimus/Prednisolone in high- risk group was effective and well-tolerated. This tailored approach to immunosuppression has delivered good outcomes while minimising overall immunosuppressive burden and side effects.

## Supplementary information

**Additional file 1.**

## Data Availability

The datasets used and analysed in this study is available in ‘supplementary materials’ section and further details can be obtained from corresponding author on reasonable request.

## Abbreviations

BPAR: Biopsy-proven acute rejection
CKD-EPI: Chronic kidney disease Epidemiology Collaboration
CRF: Calculated reaction frequency
DBD: Donation after brainstem death
DCD: Donation after circulatory death
